# Development of a Microsoft Excel tool for one-parameter Rasch model of continuous items: an application to a safety attitude survey

**DOI:** 10.1186/s12874-016-0276-2

**Published:** 2017-01-10

**Authors:** Tsair-Wei Chien, Yang Shao, Shu-Chun Kuo

**Affiliations:** 1 Medical Research Department, Chi-Mei Medical Center, Tainan, Taiwan; 2 Department of Hospital and Health Care Administration, Chia-Nan University of Pharmacy and Science, Tainan, Taiwan; 3 Department of Electronics and Information Engineering, Tongji Zhejiang College, Jiaxing, China; 4 Department of Ophthalmology, Chi-Mei Medical Center, Yong Kang Tainan City, Taiwan; 5 Department of Optometry, Chung Hwa University of Medical Technology, Jen-Teh Tainan City, Taiwan; 6 Chi-Mei Medical Center, No. 901, Chung Hwa Road, Yung Kung Dist, Tainan 710 Taiwan

**Keywords:** Rasch model, Continuous item response, Wright’s map, KIDMAP, Visual basic for application

## Abstract

**Background:**

Many continuous item responses (CIRs) are encountered in healthcare settings, but no one uses item response theory’s (IRT) probabilistic modeling to present graphical presentations for interpreting CIR results. A computer module that is programmed to deal with CIRs is required.

To present a computer module, validate it, and verify its usefulness in dealing with CIR data, and then to apply the model to real healthcare data in order to show how the CIR that can be applied to healthcare settings with an example regarding a safety attitude survey.

**Methods:**

Using Microsoft Excel VBA (Visual Basic for Applications), we designed a computer module that minimizes the residuals and calculates model’s expected scores according to person responses across items. Rasch models based on a Wright map and on KIDMAP were demonstrated to interpret results of the safety attitude survey.

**Results:**

The author-made CIR module yielded OUTFIT mean square (MNSQ) and person measures equivalent to those yielded by professional Rasch Winsteps software. The probabilistic modeling of the CIR module provides messages that are much more valuable to users and show the CIR advantage over classic test theory.

**Conclusions:**

Because of advances in computer technology, healthcare users who are familiar to MS Excel can easily apply the study CIR module to deal with continuous variables to benefit comparisons of data with a logistic distribution and model fit statistics.

**Electronic supplementary material:**

The online version of this article (doi:10.1186/s12874-016-0276-2) contains supplementary material, which is available to authorized users.

## Background

A great deal of work has been devoted to the probabilistic modeling of Likert-type responses in the past 50 years [1]. Questionnaires built and tested using item response theory (IRT) are common in educational assessment, and they are being used more frequently in health assessment [2]. However, collecting continuous responses is more often and prevalent in healthcare settings than is collecting categorically ordered data such as questionnaire surveys.

Many researchers have proposed IRT-based models of continuous item responses to deal with those multivariate behavioral research in real world [3–9], no one has provided user-friendly healthcare software for assessing, for example, pain intensity [10] or personality [4, 11]. We were, therefore, interested in developing a Microsoft Excel-based computer module that executes continuous observations similar to easily deal with binary and polytomous scores based on IRT modeling [12, 13].

Samejima [6] developed a unidimensional latent trait model for continuous responses, but it did not aim at “specifically objective” comparisons of persons and items, which is a key and unique feature of Rasch models [14]. Behar [11] applied the normal ogive (logistic curve) case of the continuous response model to items from a personality inventory. Muller [3] proposed a continuous rating scale model, based on Andrich’s [15] rating scale model (RSM) for categorical responses, which belongs to the Rasch family and so has the interesting specific objectivity property. Although Muller’s model is limited to an “integer” response format, observations can be presented for Rasch analysis in the form of a range of percentages (0–100) without decimal values. Ferrando [4] proposed a similar idea of a truncation mechanism using R language software: extending the linear response model to a nonlinear congeneric model that takes the bounded nature of the data into account rather than rescaling a latent response to fit the response format. This study creates a model in MS Excel that will take the bounded nature of the data into account and rescale a latent response to fit the response format. A unidimensional latent trait model for continuous responses that is easy and friendly use for ordinary practitioners is thus required.

There are two forms for continuous and percentage observations [16]: (i) they are very rarely exist in additive, such as continuous form (e.g., weight and height) and allow us using standard statistical techniques to make direct comparisons between groups; and (ii) they are frequently seen in the real world and might not be directly additive (e.g., time to perform a task and weight lifted with the left hand) or follow a logistic distribution. Their implications in the specific context are unlikely to be additive until they have been transformed to a linear interval score, for instance, using Rasch modeling technique.

IRT-based Rasch probabilistic modeling is a way to transform ordinal scores into interval Rasch measures [2, 3, 9, 12–16], but this type of transformation applies only to discretely ordered category scores. A computer module that is required to deal with continuous item responses (CIRs) that are often seen in healthcare settings. Accordingly, we are interested in rescaling CIRs in a percentage range from 0 to 1 using Rasch probability theory to program a Microsoft Excel-based computer module.

The first section of this article presents the designed response mechanism using the Newton-Raphson method, from which the parameters of items and persons can be estimated and well calibrated. Some resulting estimations yielded by the author-made computer module are verified with the professional Rasch Winsteps software [17] on two discretely ordered category scales [12, 13]. The second applies the model to real healthcare data and shows CIR analysis is more advantageous than is classic test theory (CTT) because the CIR uses probabilistic modeling to deal with continuous and percentage observations.

Based on the study motivation provided, this study aims to accomplish the following:

i. to verify the CIR that can be a tool used in healthcare settings.
ii. to apply the CIR to a safety attitude survey using the model’s fit statistics.
iii. to demonstrate an online computer adaptive testing (CAT) of safety attitudes for collecting data from hospital employees.

## Methods

### Module design for the continuous item responses (CIRs)

**Fig. 1:**
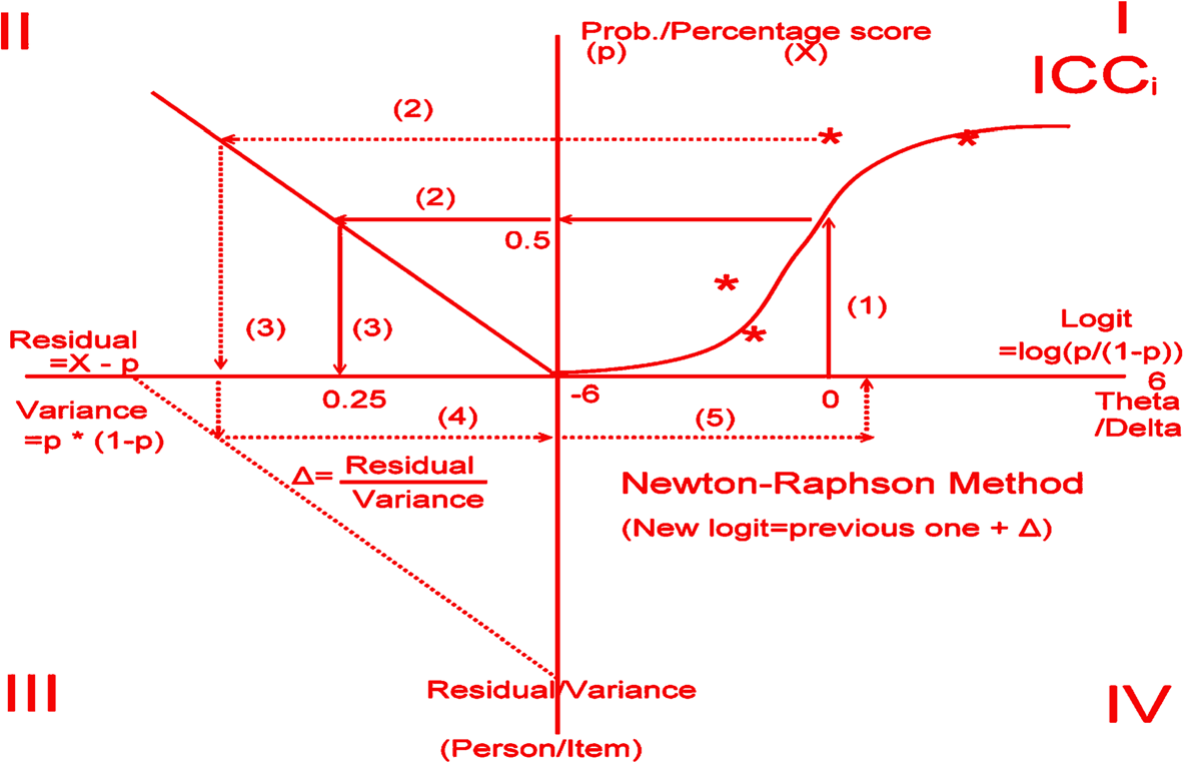
Four quadrants to show the calculation process of the continuous item responses
iii) Computing variances (*Var*_*nJ*_ = *p*_*nj*_ × (1 − *p*_*nj*_)) and residuals (*R*_*nj*_ = *X*_*nJ*_ − *p*_*nj*_) for each response (see Quadrant II in Fig. 1).
iv) Estimating the provisional *θ* for the row examinees $\left( = \mathrm{previous}\ \vartheta +{\displaystyle \sum_{j=1}^L{R}_{nj}/}{\displaystyle \sum_{j=1}^LVa{r}_{nj}}\right)$ and *δ* for the column items $\left( = \mathsf{previous}\ {\delta}_{\mathit{\mathsf{j}}}-{\displaystyle \sum_{\mathit{\mathsf{n}}=\mathsf{1}}^{\mathit{\mathsf{N}}}{\mathit{\mathsf{R}}}_{\mathit{\mathsf{n}}\mathit{\mathsf{j}}}/}{\displaystyle \sum_{\mathit{\mathsf{n}}=\mathsf{1}}^{\mathit{\mathsf{N}}}\mathit{\mathsf{V}}\mathit{\mathsf{a}}{\mathit{\mathsf{r}}}_{\mathit{\mathsf{n}}\mathit{\mathsf{j}}}}\right)$ using the Newton-Raphson Method (see Quadrants III and IV in Fig. 1), whereas the mean of *δ*_*j*_ is set at zero (i.e., ${\displaystyle \sum_{\mathit{\mathsf{j}}=\mathsf{1}}^{\mathit{\mathsf{L}}}{\delta}_{\mathit{\mathsf{j}}}=\mathsf{0}}$) [1].
v) Yielding a newly revised expectation (exp) for each response according the Rasch probability equation [i.e., $\mathsf{Prob} = \mathsf{exp}\left({\vartheta}_{\mathit{\mathsf{n}}}-{\delta}_{\mathit{\mathsf{j}}}\right)/\left(\mathsf{1} + \mathsf{exp}\left({\vartheta}_{\mathit{\mathsf{n}}}-{\delta}_{\mathit{\mathsf{j}}}\right)\right)$ and then doing the loop through the iteration process from Quadrant II through IV in Fig. 1 until reaching the minimized (Min) sum square residuals (i.e., Min ${\displaystyle \sum_{\mathit{\mathsf{n}}=\mathsf{1}}^{\mathit{\mathsf{N}}}{\displaystyle \sum_{\mathit{\mathsf{j}}=\mathsf{1}}^{\mathit{\mathsf{L}}}\left({\mathit{\mathsf{O}}}_{\mathit{\mathsf{n}}\mathit{\mathsf{j}}}-{\mathit{\mathsf{E}}}_{\mathit{\mathsf{n}}\mathit{\mathsf{j}}}\right)}}$ at a level of 0.05, 0.01, or less.

Using Microsoft Excel VBA (Visual Basic for Applications), we designed a computer module that minimizes the residuals (i.e., Min ${\displaystyle \sum_{n=1}^N{\displaystyle \sum_{j=1}^L\left({O}_{nj}-{E}_{nj}\right)}}$, where *N* = sample size, L = item length, O = observed score, and E = expected value) for estimating item difficulties parameters (*δ*_*j*_) and person ability (*ϑ*_*n*_). The five module steps are illustrated below:

i) Rescaling the variable scores to a percentage response [i.e., recorded response = (observed response − minimum response)/(maximum response − minimum response)], and setting both initial *ϑ*_*n*_ and *δ*_*j*_ = 0 in a logit unit. This is, at the origin of the measurement scale, every person is estimated to be at the same level of ability, and each item is estimated to be equally difficult.
ii) Calculating the initial expected values for each response using the one-parameter Rasch probability (Prob) equation: $\mathrm{Prob} = \exp\ \left({\mathit{\mathsf{\theta}}}_{\mathit{\mathsf{n}}}-{\delta}_j\right)/\left(1 + \exp\ \left({\mathit{\mathsf{\theta}}}_{\mathit{\mathsf{n}}}-{\delta}_j\right) = \exp\ \left(0-0\right)/\left(1 + \exp\ \left(0-0\right)\right) = 1/\left(1 + 1\right)\right) = 0.5$ (see Quadrant I in Fig. 1).

### Rasch fit statistics used in this study

In addition to the person measures and item difficulties estimated in our proposed computer module, Rasch mean square (MNSQ) fit statistics (consisting of both item index and person INFIT and OUTFIT indices) describe the fit to the model. The MNSQ fit statistics have a χ^2^ distribution and an expected value of 1, where fit statistics greater than 1 can be interpreted as demonstrating that the observed scores are aberrant distortions of (or underfit) the model’s expectation of unidimensionality [18]. Conversely, fit statistics less than 1 can be explained as items (or persons) that overfit the model, or that are highly correlated (i.e., item loading in an explorative factor analysis) with the summation score.

The INFIT MNSQ is referred to as the weighted MNSQ and OUTFIT MNSQ is referred to the unweighted MNSQ. Both are derived from the squared standardized residuals [${\mathit{\mathsf{Z}}}_{\mathit{\mathsf{n}}\mathit{\mathsf{j}}}=\sqrt{{\mathit{\mathsf{R}}}_{\mathit{\mathsf{n}}\mathit{\mathsf{j}}}^{\mathsf{2}}/\mathit{\mathsf{V}}\mathit{\mathsf{a}}{\mathit{\mathsf{r}}}_{\mathit{\mathsf{n}}\mathit{\mathsf{j}}}}$, whereas *Var*_*nj*_ are response variances, and the person (n) and item (j) OUTFIT MNSQs are presented as ${\displaystyle \sum_{\mathit{\mathsf{j}}=\mathsf{1}}^{\mathit{\mathsf{L}}}{\mathit{\mathsf{Z}}}_{\mathit{\mathsf{n}}\mathit{\mathsf{j}}}^{\mathsf{2}}/\mathit{\mathsf{L}}}$ and ${\displaystyle \sum_{\mathit{\mathsf{n}}=\mathsf{1}}^{\mathit{\mathsf{N}}}{\mathit{\mathsf{Z}}}_{\mathit{\mathsf{n}}\mathit{\mathsf{j}}}^{\mathsf{2}}/\mathit{\mathsf{N}}}$] for each item/person interaction. The OUTFIT MNSQ is the average of the standardized residual variance across items and persons; it is unweighted, which means that the estimate produced is relatively more affected by unexpected responses distant to item or person measures [19]. For the INFIT MNSQ the residuals are weighted by their individual variance [i.e., $\frac{{\displaystyle \sum_{\mathit{\mathsf{n}}=\mathsf{1}}^{\mathit{\mathsf{N}}}{\left(\mathit{\mathsf{R}}\mathit{\mathsf{n}}\mathit{\mathsf{j}}\right)}^{\mathsf{2}}}}{{\displaystyle \sum_{\mathit{\mathsf{n}}=\mathsf{1}}^{\mathit{\mathsf{N}}}\mathit{\mathsf{V}}\mathit{\mathsf{n}}\mathit{\mathsf{j}}}\mathit{\mathsf{i}}}$ for the item (j) and $\frac{{\displaystyle \sum_{\mathit{\mathsf{j}}=\mathsf{1}}^{\mathit{\mathsf{L}}}{\left(\mathit{\mathsf{R}}\mathit{\mathsf{n}}\mathit{\mathsf{j}}\right)}^{\mathsf{2}}}}{{\displaystyle \sum_{\mathit{\mathsf{j}}=\mathsf{1}}^{\mathit{\mathsf{L}}}{\mathit{\mathsf{V}}}_{\mathit{\mathsf{n}}\mathit{\mathsf{j}}}}}$ for the person (n)] to minimize the effect of unexpected responses far from the measure.

The INFIT MNSQ is relatively more affected by unexpected responses closer to item and person measures [18]. In this study, the INFIT MNSQs for items less than 1.5 means data fitting to the Rasch model of the unidimensionality requirement and the OUTFIT MNSQs for persons less than 2.0 indicates person responses without aberrant or careless behaviors [20]. Additionally, the squared standardized residuals $\left({\mathit{\mathsf{Z}}}_{\mathit{\mathsf{n}}\mathit{\mathsf{j}}}=\sqrt{{\mathit{\mathsf{R}}}_{\mathit{\mathsf{n}}\mathit{\mathsf{j}}}^{\mathsf{2}}/\mathit{\mathsf{V}}\mathit{\mathsf{a}}{\mathit{\mathsf{r}}}_{\mathit{\mathsf{n}}\mathit{\mathsf{j}}}}\right)$ for each response less than 2.0 (i.e., *p* < 0.05) is defined as a nonsignificant deviation from the model’s expected value.

### Validation of the CIR model

Besides the aforementioned theoretical modelling method, two ordered-category datasets derived using the 11-item dichotomous and 26-item polytomous scales [12, 13] were further used to verify whether our proposed computer module yield accurate results in measures, difficulties, and fit statistics similar to those of the professional Rasch Winsteps software [17]. The percentage scores were rescaled to a range between 0 and 1.0. For instance, the only two values of 0 and 1.0 were assigned the original codes of 0 and 1, and the 5-point Likert-type scaling scores were uniformly transformed to 0, 0.25, 0.5, 0.75, and 1.0, respectively.

The 2-item variables downloaded from a 2015 US NBA game between GSW (Golden State Warriors) and OKC (Oklahoma City Thunder) on Feb. 27, 2016 were used for showing the CIR that transforms raw scores into Rasch estimated logits.

### Demonstrations of real healthcare data using the Rasch CIR model

Two published papers [21, 22] were referenced to evaluate whether our proposed Rasch CIR model using the fit statistics and the expected responses is more beneficial than the traditional CTT method because it discloses more valuable messages. One data source was collected from a Taiwan hospital in a safety attitude survey [22, 23]. An online computer adaptive testing (CAT) that can deal with many CAT procedurals on each domain in a test was demonstrated to collect safety attitude data from hospital employees.

## Statistical analysis

In the author-made CIR module, R^2^ was calculated using the regression equation $\left(={\displaystyle \sum_{\mathit{\mathsf{n}}=\mathsf{1}}^{\mathit{\mathsf{N}}}{\displaystyle \sum_{\mathit{\mathsf{j}}=\mathsf{1}}^{\mathit{\mathsf{L}}}{\left({\mathit{\mathsf{E}}}_{\mathit{\mathsf{n}}\mathit{\mathsf{j}}}-\overline{{\mathit{\mathsf{X}}}_{\mathit{\mathsf{i}}\mathit{\mathsf{j}}}}\right)}^{\mathsf{2}}}}/{\displaystyle \sum_{\mathit{\mathsf{n}}=\mathsf{1}}^{\mathit{\mathsf{N}}}{\displaystyle \sum_{\mathit{\mathsf{j}}=\mathsf{1}}^{\mathit{\mathsf{L}}}{\left({\mathit{\mathsf{X}}}_{\mathit{\mathsf{n}}\mathit{\mathsf{j}}}-\overline{{\mathit{\mathsf{X}}}_{\mathit{\mathsf{i}}\mathit{\mathsf{j}}}}\right)}^{\mathsf{2}}}}\right)$. The Paired-*t* and independent-*t* tests for two variables were used to compare the raw scores, percentage scores, and Rasch expected response scores using the 2-variable dataset of safety attitudes. The person OUTFIT MNSQ was obtained using the two values of standardized residual (Z) and item length (L) in the equation $\left(={\displaystyle \sum_{\mathit{\mathsf{j}}=\mathsf{1}}^{\mathit{\mathsf{L}}}{\mathit{\mathsf{Z}}}_{\mathit{\mathsf{n}}\mathit{\mathsf{j}}}^{\mathsf{2}}/\mathit{\mathsf{L}}}\right)$. The Wright Map (or named person-item map) [24] and the KIDMAP [25] were drawn using the CIR module.

## Results

### Validation of the Rasch CIR model

**Fig. 2:**
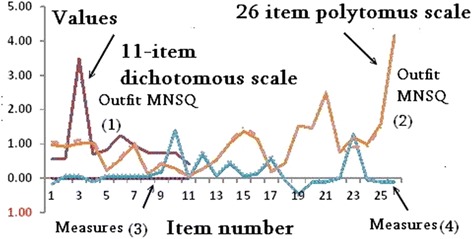
Four combinations of Outfit MNSQ and measures on two scales are closely overlaid

The Outfit MNSQ statistics and measures yielded by the proposed CIR computer module and by the Rasch professional Winsteps software [17] on the two study scales [12, 13] were equivalent (Fig. 2). We can see that spot lines of the CIR and Winsteps software in 4 combinations of Outfit MNSQ and measures on two scales (i.e., dichotomous and polytomous responses) are closely overlaid [26].

**Fig. 3:**
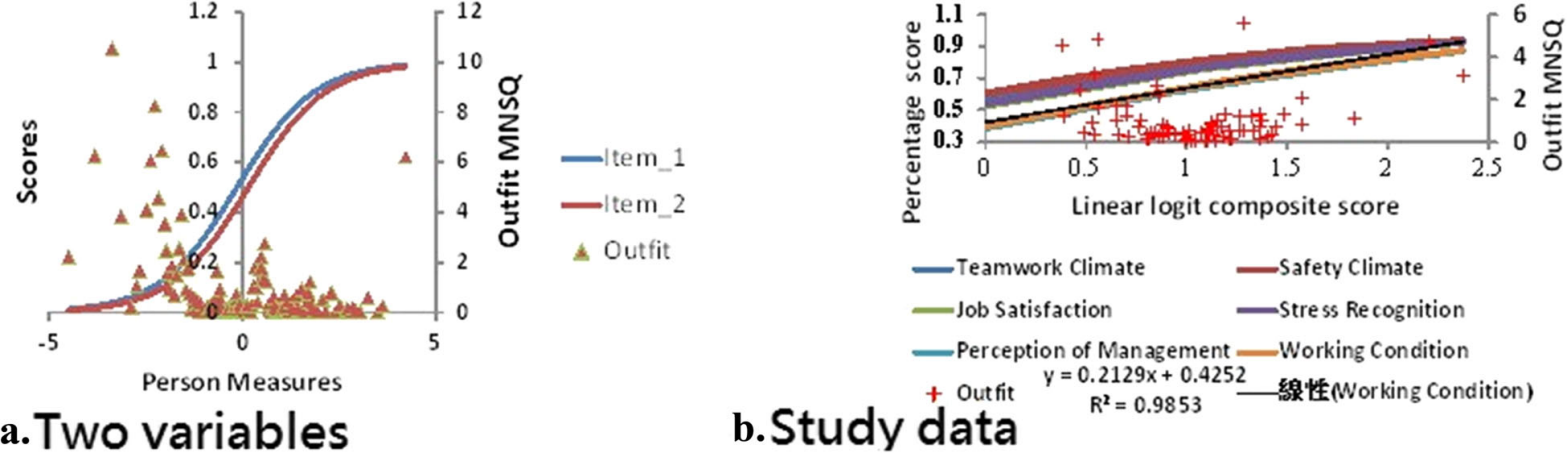
Case studies of continuous item responses for 2-item competition basketball scores (*left*) and 6 domain ﻿scores to a safety attitude survey (*right*)

The CIR was applied to the 2-item competition basketball scores between GSW and OKC on Feb. 27, 2016. Two variables’ item character curves (ICC) with person Outfit MNSQ are present in Fig. 3 (left panel) after transforming raw scores into linear logits. It can be evident of the fact that logit scores (on X axle) are more appropriate than those raw scores (on Y axle) when adding them for group comparisons and release more information, such as person Outfit MNSQ, to readers on a plot. The more difficult items (i.e., lower scores) are shown to the right-hand side. The more aberrant responses (i.e., response pattern not consistent to others) are shown to the top with Outfit MNSQ greater than 2.0 [20] on a plot (left panel in Fig. 3).

### Real data used to release information with visual presentations

Besides the theoretical modelling method verified in Fig. 1, the accurate measures estimated proved in Fig. 2, and the logit scores transformed by the CIR in Fig. 3 (on X axle), we used the survey resulting data from a Taiwan medical center with 1200 beds in 2015 to demonstrate the application of the CIR t (see Additional file 1 in Appendix). A total of 80 departments were included to show the 6 domain scores (including Teamwork Climate, Safety Climate, Job Satisfaction, Stress Recognition, Perception of Management, and Working Condition) [21, 22, 27, 28] on a plot (right panel in Fig. 3). All domain scores are above a percentage of 0.3 (equivalent to 30 points in a 0–100 scale), two domains (i.e., Perception of Management and Working Condition, not present a linear pattern with a R-square around 0.98) are more difficult than other four, and several aberrantly unexpected responses are present in Figure (right) with Outfit MNSQ greater than 2.0 [20].

The data metric of 80 units * 6 domains were analyzed with the model-data fit (see Additional file 2 in Appendix). R^2^ was 0.65 with F = 1.96 (*p* = 0.18) using the regression equation $\left(={\displaystyle \sum_{\mathit{\mathsf{n}}=\mathsf{1}}^{\mathit{\mathsf{N}}}{\displaystyle \sum_{\mathit{\mathsf{j}}=\mathsf{1}}^{\mathit{\mathsf{L}}}{\left({\mathit{\mathsf{E}}}_{\mathit{\mathsf{n}}\mathit{\mathsf{j}}}-\overline{{\mathit{\mathsf{X}}}_{\mathit{\mathsf{i}}\mathit{\mathsf{j}}}}\right)}^{\mathsf{2}}}}/{\displaystyle \sum_{\mathit{\mathsf{n}}=\mathsf{1}}^{\mathit{\mathsf{N}}}{\displaystyle \sum_{\mathit{\mathsf{j}}=\mathsf{1}}^{\mathit{\mathsf{L}}}{\left({\mathit{\mathsf{X}}}_{\mathit{\mathsf{n}}\mathit{\mathsf{j}}}-\overline{{\mathit{\mathsf{X}}}_{\mathit{\mathsf{i}}\mathit{\mathsf{j}}}}\right)}^{\mathsf{2}}}}\right)$. The *t*-value of the two item difficulties (−0.23 and −0.42 logits for Teamwork Climate and Safety Climate) was $\mathsf{0.48}\left(=\left(\hbox{-} \mathsf{0.23}\hbox{-}\ \hbox{-} \mathsf{0.42}\right)/\sqrt{\mathsf{1}/\mathsf{13.76}+\mathsf{1}/\mathsf{12.39}}\Big),\mathsf{p}=\mathsf{0.63}\right)$, not significantly different from each other using the traditional *t*-test.Additional file 2 Video demonstration regarding application of the Excel CIR module http://www.healthup.org.tw/marketing/course/information/rasch_cir.mp4. (MP4 45229 kb)


**Fig. 4:**
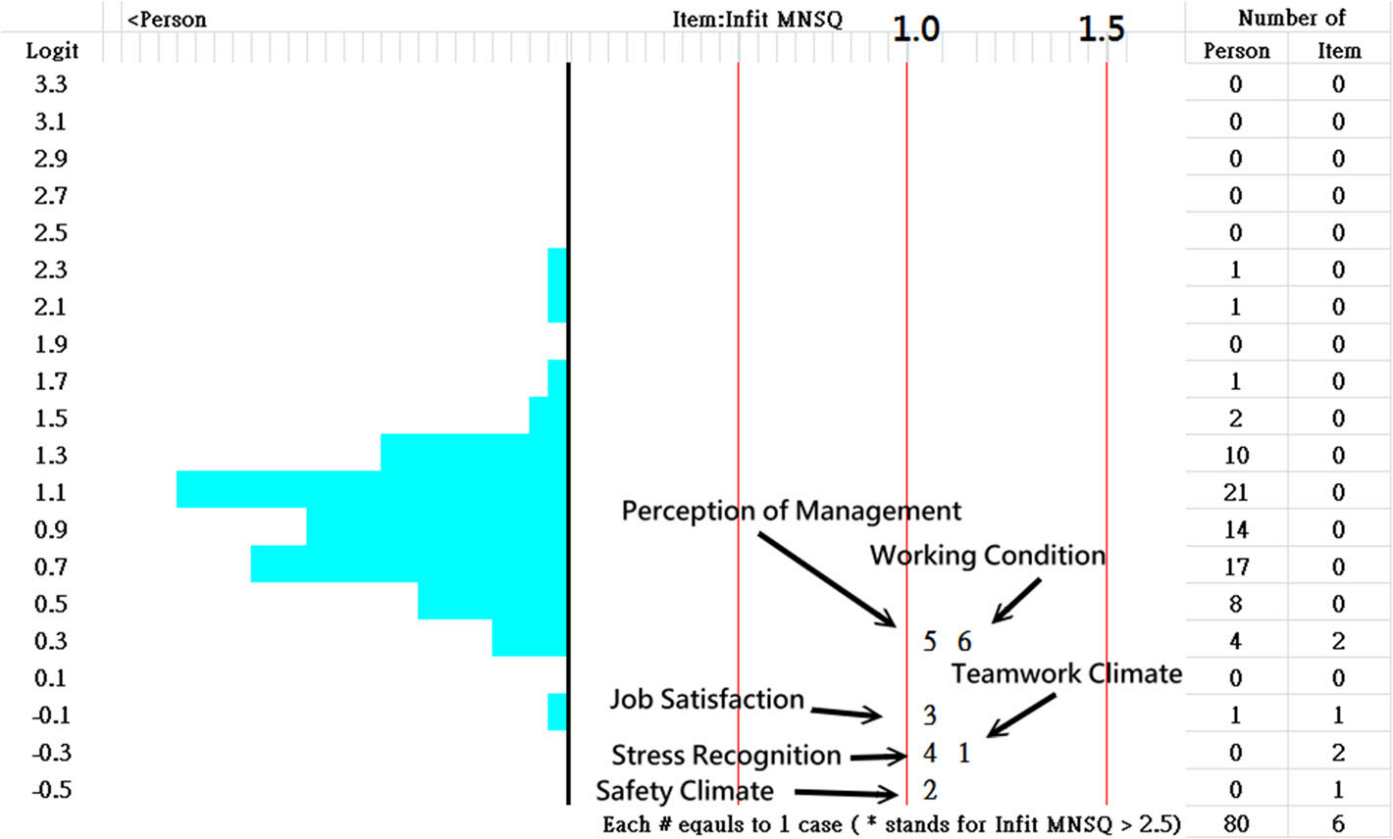
Wright map shows person and item dispersions

A Wright map shows that the 6 domains and a single construct when referring to INFIT MNSQs <1.5; the person distribution is also shown (Fig. 4, left).

**Fig. 5:**
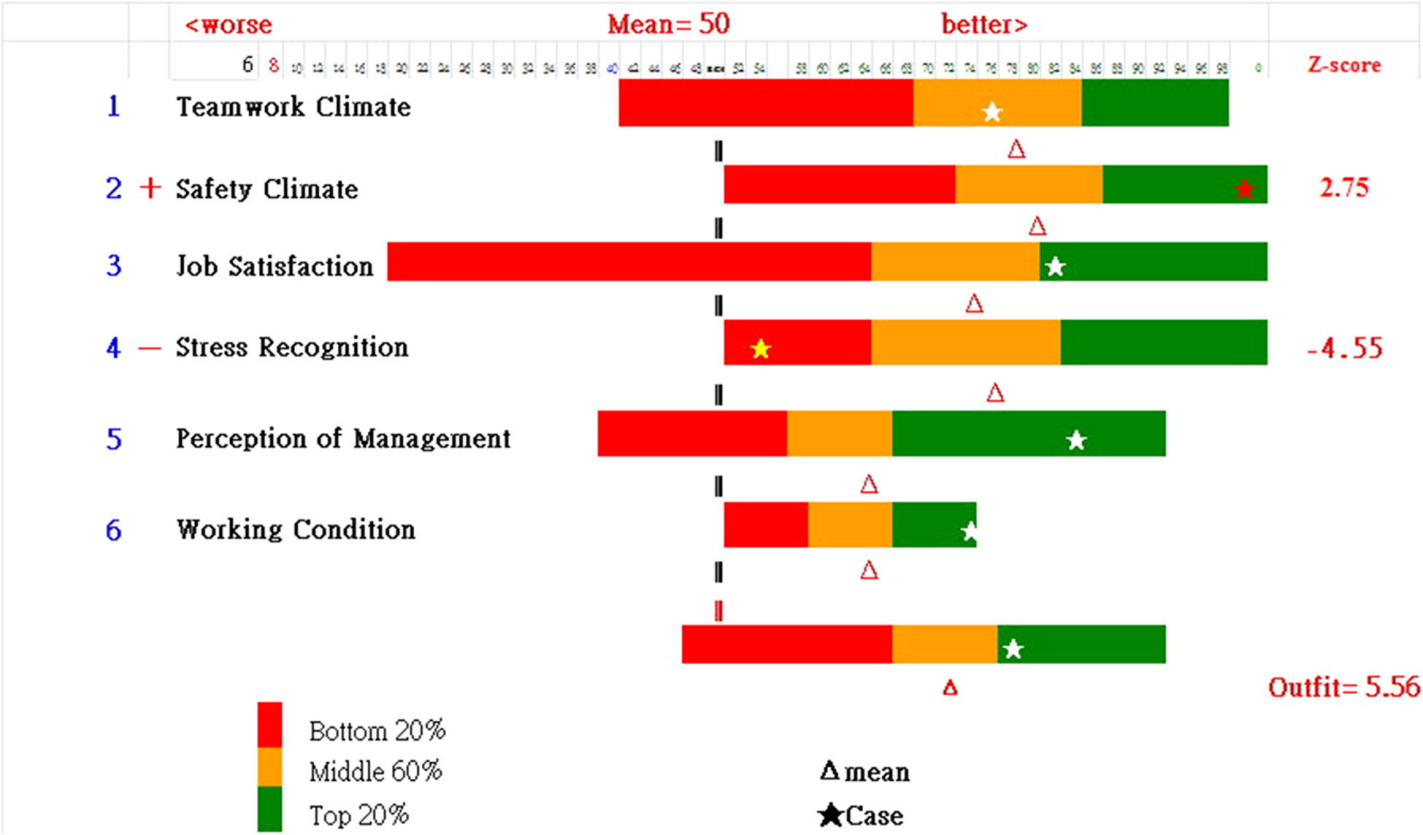
Box-plot KIDMAP shows a person performance on a sheet

The results of a case department show an aberrant OUTFIT MNSQ of 5.67 (>2.0) in the Box KIDMAP (Fig. 5, right bottom). One domains—Safety Climate—shows a significantly high Z-score (=2.75): > 2.0, and another dimension—Stress Recognition—shows a significantly low Z-score (= − 4.55): < −2.0, implying the strength and weak comparing to other counterparts.

From the Box KIDMAP we also see that the most difficult dimension (i.e., the one with the lowest mean score) was Perception of Management and Working Condition. The easiest one (the one with the highest mean scores) was Safety Climate. Most of the dimension scores appear to be normally distributed, but Job Satisfaction was slightly negatively distributed.

**Fig. 6:**
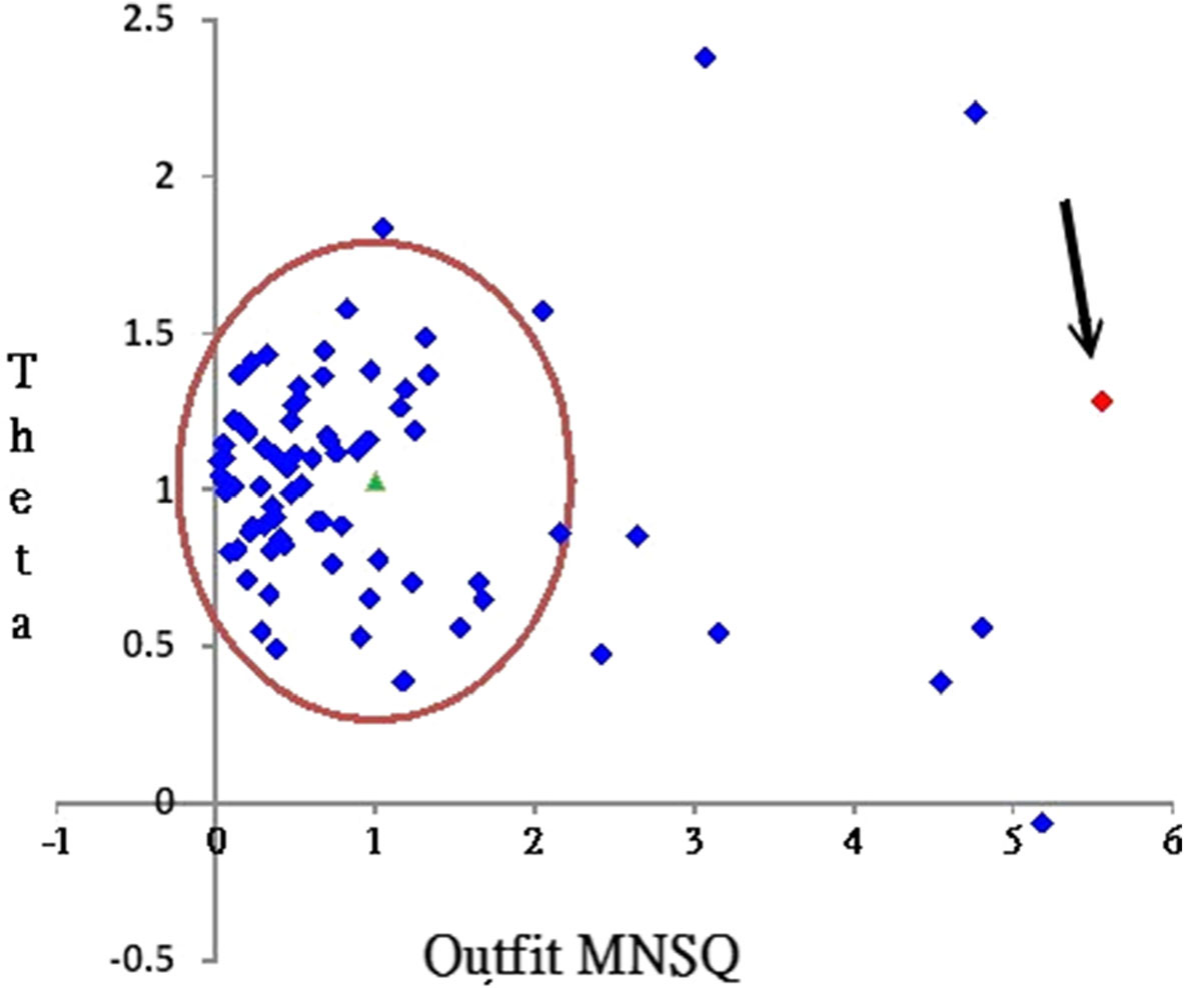
A person performance referred to the overall counterparts

The most unexpected misfit case—an OUTFIT MNSQ 5.56 (>2.0)—was extracted at the 1.28 logits point (Fig. 6). We can see the aberrant response pattern with a high OUTFIT MNSQ (>2.0) (e.g., this anomaly might be most attributable to the two domains with the two Z-scores of 2.75 and −4.55 in Fig. 5).

### An online computer adaptive testing (CAT) was demonstrated

**Fig. 7:**
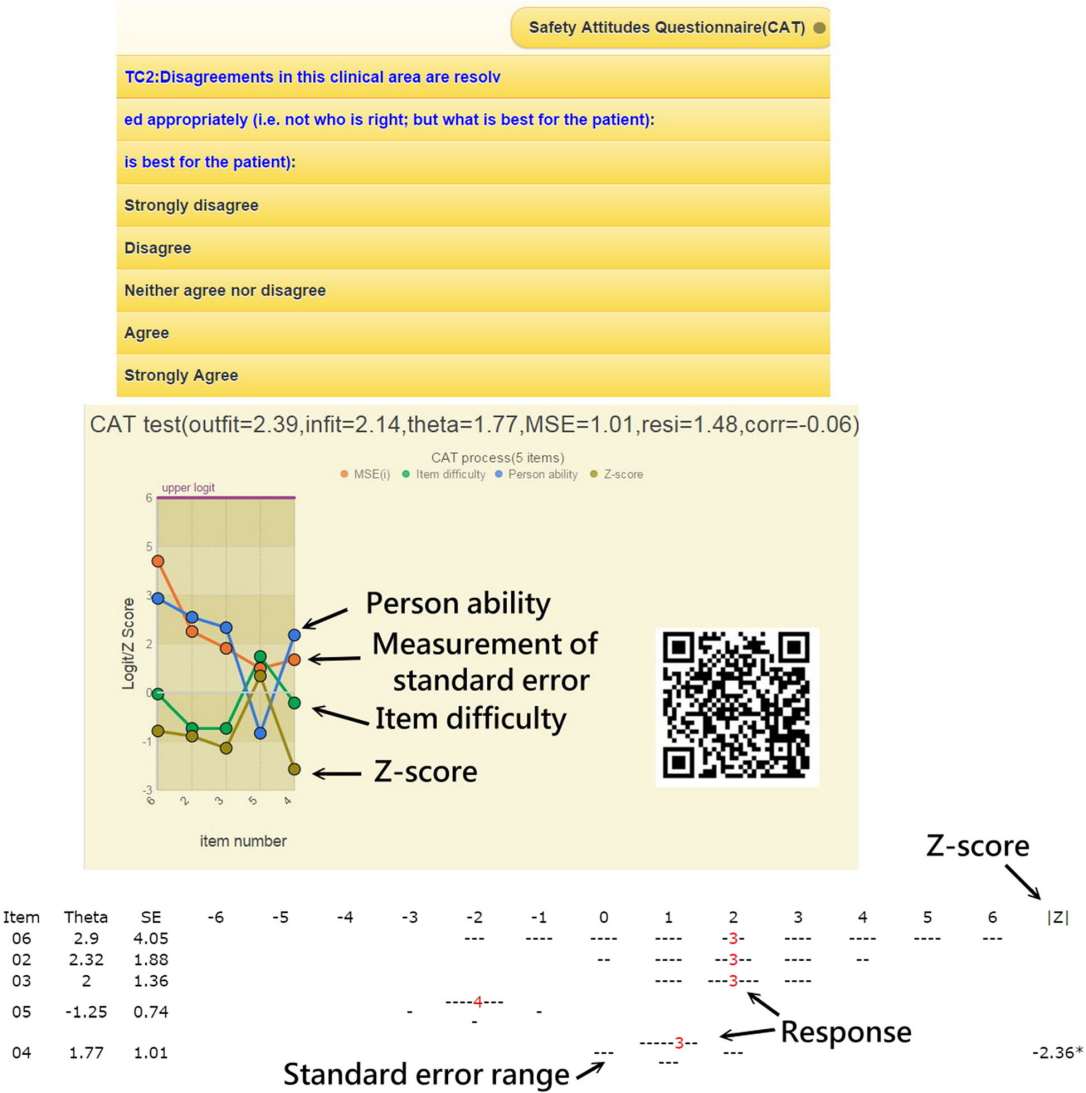
A snapshot of the CAT process

By scanning the QR code (Fig. 7, middle right), the CAT icon appears on the patient’s mobile phone. We demonstrate an alternative way for gathering respondent’s safety attitudes with item-by-item in action (see Additional file 3 in Appendix). Person fit (ie, infit and outfit mean square) statistics showed the respondent behaviors. Person theta is the provisional ability estimated by the online CAT module.Additional file 3 Computerized adaptive testing of the patient safety survey on six subscales http://www.healthup.org.tw/marketing/course/information/safetysurveyKREA.mp4. (MP4 14747 kb)

The MSE (e.g., measurement of standard error) in Fig. 7 was generated by the following formula [29]: 1/√(Σ variance(*i*)), where *i* refers to the CAT finished items responded to by a person [30]. The residual (resi) on top in Fig. 7 was the average of the last three change differences between the pre-and post-estimated abilities on each CAT step. CAT will stop if the residual value is less than 0.05, indicating a stable estimation in process. Corr refers to the correlation coefficient between the CAT estimated measures and its step series numbers using the last three estimated theta (= person measure) values. The flatter the theta trend, the higher the probability that the person measure is convergent with a final estimation.

## Discussion

### Principal findings

Our most important finding was that the OUTFIT MNSQ and person measures yielded by our proposed CIR module were not significantly different from the professional Rasch Winsteps software on the two dichotomous and polytomous scales. Our second most important finding was that the probabilistic modeling of our CIR module presented readers much more valuable messages (e.g., fit statistics and visual representations of a Wright map and KIDMAP) and illustrated the CIR module’s advantage over CTT, especially when using the expected scores (instead of the raw scores) in comparing group (or item) differences.

A video regarding the CIR application is demonstrated in a MP4 format at reference [31].

There are so many comparative scores that are not, or might not be, additive but that follow a logistic distribution in the real world [16]. The Rasch model formulates a nonlinear relationship between nonlinear raw scores and additive measures [31]. Figure 4 shows that we were more concerned about locating that person on the true score trait (on X axle) than in having that person obtains a raw score (on Y axle). This is why we preferred to use the model-expected scores for comparison in Fig. 1. Unfortunately, we have not seen a statistical modeling technique that can deal with CIRs as we did using our Microsoft Excel module.

### Implications and future considerations

We showed that fit statistics and visual representations of a Wright map and KIDMAP offer much more valuable messages to readers. The premises consist of the model-expected scores that should first be yielded by Rasch analysis. Fit statistics are thus produced, and the Wright map and KIDMAP follow. An adequate CIR computer routine is thus required.

Using our proposed module in the future will not be limited to the two types of continuous item responses (i.e., two basketball completion scores and six items of the patient safety survey shown in Fig. 4) illustrated here. Scores for a single item (e.g., serum levels of liver enzymes and lipids [32]) can also be easily transformed into Rasch interval logit measures used for statistical group comparison or product moment correlation analysis in practice.

Our CIR computer program can be simultaneously used for many between-test dimension scores in a survey, like in this study. For example, the Clinical Dementia Rating scale [33] used for patients with dementia consists of six domains. We can present the Wright map and KIDMAP to the patient or their family proxy in the same way that educational performance sheets are presented to students or their parents [24, 25].

### Strengths

All Rasch measurement analysis software is used for dichotomous and polytomous ordered category scales [33, 34]. In the healthcare field, there are so many continuous variables that cannot be analyzed and plotted a Wright map and KIDMAP. Many studies [3–9] have proposed IRT-based models used for CIRs but have not provided user-friendly software such as MS Excel version in this study. We demonstrated one example of a safety attitude survey using continuous variables analyzed with a user-friendly MS Excel module to plot a Rasch Wright map and KIDMAP.

The Wright map was improved by adding the item INFIT MNSQ dispersion on the right (see Fig. 4) and with color features, which has never before been used to interpret the unidimensionality feature together with person distribution in a diagram.

The KIDMAP has evolved to a colorful item-by-item bar-chart plot (see Fig. 5) different from the traditional monochrome diagram [35, 36–38] or KIDMAP solely for dichotomous scales [25]. More information has been included in the colorful KIDMAP: all person distributions and dispersions along all items, Z-scores displaying the most unexpected items, and the individual person location points on items suitable for polytomous and continuous variables. A person performance referred to the overall counterparts is also present in a scatter plot (Fig. 6) that can easily select aberrant respondents for further improvement and concerns with respect to the example of hospital safety attitude survey.

It is worth mentioning that the plot combined model-expected item character curves with OUTFIT MNSQs in a diagram (Fig. 3) which is featured and outlined together to present many valuable messages that are not easily disclosed using the traditional CTT method. The between-test score (i.e., CIR format) comparison demonstrated in this study is an extension of those within-test score analyses (i.e., ordered category format). For instance, we concern with outcomes between groups (with continuous format) after gathering data on the individual base (with ordered category format).

Many studies [39–43] have reported that item response theory (IRT)-based computer adaptive testing (CAT) has the advantages of both long-form and short-form questionnaires [44–46] in precision and efficiency. Many employees already own mobile phones. No studies reported online CAT via mobile phones in medical fields, especially implementing multiply-domain online CAT together in a test. We demonstrated it for gathering survey data regarding safety attitudes here (See Fig. 7).

### Limitations

This study has at least four limitations. First, the residuals in person-item interaction cells are significantly small in the CIR format scenario. A normalization procedure for letting the mean of INFIT and OUTFIT MNSQ = 1.0 is necessary for easy interpretation by readers, assuming that the acceptable criterion is between 0.5 and 1.5 required by the Rasch model.

Second, IRT parameters in the online CAT example is retrieved from a published paper [21] using the IRT graded response model (GRM) [47], which did not comply with “specifically objective” comparisons of persons and items as the unique feature of Rasch models [14]. We can replace all the discrimination parameters in GRM with 1 to fit Rasch model’s requirement in future study.

Third, we combined the person OUTFIT MNSQ into the model-expected item character curves in a diagram (Fig. 3) because two vertical axles with a significant difference distort the diagram and make it difficult to interpret without training, but easy to compare the two vertical axles after training.

Fourth, the CIR module is easy to yield greater Outfit MNSQ in the extreme two sides (i.e., toward zero and 100 percentage score) because of minimal variance leading a higher value of person Outfit MNSQ $\left(={\displaystyle \sum_{\mathit{\mathsf{j}}=\mathsf{1}}^{\mathsf{L}}{\mathit{\mathsf{R}}}_{\mathit{\mathsf{n}}\mathit{\mathsf{j}}}^{\mathsf{2}}/\mathit{\mathsf{V}}\mathit{\mathsf{a}}{\mathit{\mathsf{r}}}_{\mathit{\mathsf{n}}\mathit{\mathsf{j}}}}/\mathsf{L}={\displaystyle \sum_{\mathit{\mathsf{j}}=\mathsf{1}}^{\mathit{\mathsf{L}}}{\mathit{\mathsf{Z}}}_{\mathit{\mathsf{n}}\mathit{\mathsf{j}}}^{\mathsf{2}}/\mathit{\mathsf{L}}}\right)$. For instance, a variance (=0.5*0.5) in middle point is greater than it (=0.1*.09) in both extreme sides.

Fifth, we merely emphasize the usefulness on the healthcare settings, not suggest the CIR being applied to the clinical utility. Because the chemical Lab examination scores are not like the survey items having a consistent scaling score. Whether the clinical scores should be standardized first prior to analysis is required to further explored and researched in future.

## Conclusion

Because of advances in computer technology, healthcare users who are familiar to MS Excel can easily apply the study CIR module to deal with continuous variables to benefit comparisons of data with a logistic distribution and model fit statistics.

## Additional files

Additional file 1: Microsoft Excel-based computer module for continuous item responses. (XLSM 2705 kb)

## Abbreviations

CAT: Computer adaptive testing
CIR: Continuous item responses
CTT: Classic test theory
GRM: Graded response model
ICC: Item character curves
IRT: Item response theory
MNSQ: Mean square error
MSE: Measurement of standard error
RSM: Rating scale model
VBA: Visual basic for applications
